# CYTOCON DB: A versatile database of human cell and molecule concentrations for accelerating model development

**DOI:** 10.1002/psp4.12897

**Published:** 2023-01-12

**Authors:** Rajat Desikan, Priya Jayachandran

**Affiliations:** ^1^ Clinical Pharmacology Modeling & Simulation (CPMS) GSK Stevenage UK; ^2^ Global Product Development Pfizer Inc. Cambridge Massachusetts USA

In this issue of *CPT: Pharmacometrics and Systems Pharmacology*, Leonov et al.^1^ present a powerful tool for quantitative systems pharmacology (QSP) practitioners and immune modelers: “CYTOCON DB,” a comprehensive and manually curated database of publicly available concentrations of cells, cytokines, chemokines, and other molecules in various human organs in both healthy and diseased states.

Organized information in the form of searchable databases can empower and spur innovation and even drive the progress of entire fields of research. The Research Collaboratory for Structural Bioinformatics (RCSB) Protein Data Bank (PDB)^2^ is a prime example; serving as the first open access digital repository of extensive sequence and structural information about proteins. Today, it serves the learning and experimental needs of structural biologists, computational scientists, students, and educators while catalyzing the creation of other protein structural databases, such as the “Membranome”^3^ and “AlphaFold DB,”^4^ collectively accelerating the field of protein structure and folding. Analogously, CYTOCON DB could potentially be a game‐changer for QSP immune modelers across therapeutic areas.

QSP is a cutting‐edge modeling tool that facilitates model‐informed drug discovery and development (MID3)^5^, ^6^, ^7^ and informs decision making by combining extensive mechanistic knowledge of biological pathways and physiology with multiple streams of data ranging from omics to preclinical experiments and clinical biomarkers. Immune responses are a core component of QSP models across therapeutic areas, including infectious diseases, vaccines, oncology, autoimmune disorders, and rare diseases (Figure 1). A major obstacle for developing physiologically realistic immune models is a lack of organized information about baseline (pretreatment) concentrations of various cells involved in innate and adaptive immunity (e.g., dendritic cells, macrophages, neutrophils, lymphocytes – B cells, T cells, natural killer cells), and immune‐regulatory molecules like cytokines and chemokines that serve as major factors to inform disease progression. For example, in oncology, tumor concentrations of inflammatory and regulatory cells and cytokines render the tumor as either “hot” or “cold,” which results in different progression patterns and response to therapies, and, therefore, prognosis.^8^ Similarly, with autoimmune disorders, optimizing the dose for immunomodulatory therapies necessitates the use and development of QSP models that account for differences in inflammatory cytokine levels and autoreactive immune responses between healthy individuals and patients.

**FIGURE 1:**
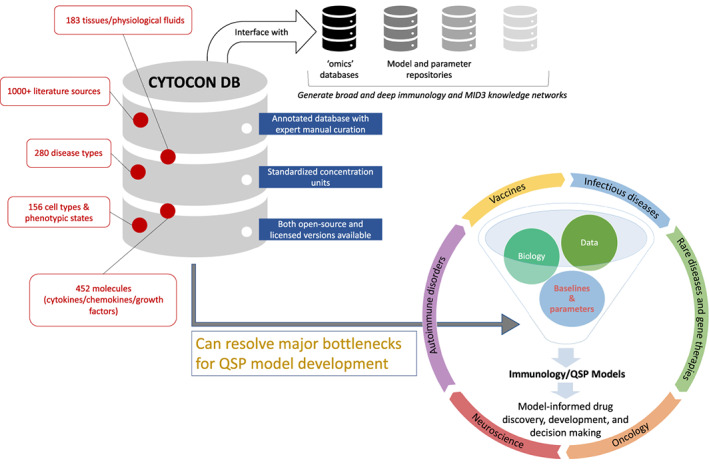
A schematic highlighting how CYTOCON DB can interface with QSP model development across therapeutic areas and facilitate accelerated MID3 approaches. CYTOCON DB can also potentiate the generation of broad and deep knowledge networks for immunology and MID3 by interfacing with complementary databases. MID3, model‐informed drug discovery and development; QSP, quantitative systems pharmacology

QSP models typically consist of tens‐to‐hundreds of state variables, parameters, equations, and initial conditions. Parameterization of such models is a laborious and rate‐limiting process that often requires extensive mining of literature followed by data extraction.^9^, ^10^ A key issue for accessing human data from literature is the enormous heterogeneity in experimental protocols and assay conditions across studies that necessitate development of domain expertise and time‐intensive manual conversion of measurements and units by QSP modelers to ensure consistency. CYTOCON DB provides a solution to this issue via an expert manual curation process that results in readily available, annotated, and organized data in standard units. Subject matter experts associated with CYTOCON DB manually mark a paper, extract data, annotate, and standardize it for inclusion into the database (see Leonov et al.^1^ for further details). Impressively, an independent expert reviews the data and corrects errors. This two‐step expert verification promises a high‐quality database. At the time of publication, CYTOCON DB covers 280 disease types, 183 tissues/physiological fluids, 452 molecules (cytokines/chemokines/growth factors), and 156 cell types and phenotypic states, all mined from nearly a thousand papers and other public sources (Figure 1). The database can be accessed as a web application via any standard browser (https://cytocon‐open.insysbio.com/) and a user‐friendly interface helps to filter queries by disease, tissue, and other qualifiers. Being a new application, while this interface is fully functional, it also presents opportunities for refinement.

From a user point‐of‐view, although this database was created by a commercial QSP modeling company – InSysBio ‐ both commercial and open‐source versions of the database are available, the latter being crucial for scientific progress. Notably, the open‐source version is updated once a year and is free for both commercial and non‐commercial purposes. Because database creation and maintenance require committed resources, revenue generation from commercial licenses as well as soft advantages like gain of reputation in the QSP community may provide ample justification to maintain the open‐source version of the database. Nonetheless, concerns regarding future accessibility and sustainability remain. Potential avenues for sustainable open‐source development of CYTOCON DB include crowdsourcing of responsibilities across community experts in industry and academia, applications for grants from funding agencies, and industry‐academia consortium models where a group of institutions and companies pay a licensing fee to ensure that the entire database remains open‐source to the community.

Future extensions and improvements to CYTOCON DB are possible. An important, orthogonal, but somewhat difficult extension of the database is to include other species commonly used in preclinical studies, such as mice, rats, and non‐human primates. Doing so could facilitate the development of QSP models that span both preclinical and clinical phases of drug development, potentially accelerating dose optimization and accurate predictions of first‐in‐human dosing in complex immunotherapeutic areas such as vaccines, infectious diseases, neuroscience, and oncology. Other possible avenues for technical improvements include developing knowledge graphs of relevant literature via automated text mining that could automatically flag relevant papers to the CYTOCON DB team for updating of the database, and use of natural language processing for complex and intelligent queries of the database. Finally, CYTOCON DB could both generate new databases, and be linked with existing databases for potentially generating both broad and deep knowledge networks in immunology and MID3. For example, “omics” databases and CYTOCON DB could be combined for linking gene expression to concentrations of various immune phenotypes across tissues and disease conditions. Similarly, repositories of existing models and published parameter values, such as BioModels^11^ (https://www.ebi.ac.uk/biomodels/) and PKPD‐AI (https://www.pkpdai.com/), can be combined with CYTOCON DB for accelerating MID3 approaches. These developments would perhaps require additional standardization of the database as well as open source availability of raw and meta data for interfacing with other tools. While requiring some effort, this process provides an opportunity for CYTOCON DB to evolve and grow more useful and versatile with time.

CYTOCON DB enters a growing field of data science that has witnessed expanded utility over the years. Through filling a void for accessible, standardized, expert‐curated data on baseline concentrations of immune cells and cytokines in human tissues, CYTOCON DB is the first of its kind and is expected to perform a crucial catalytic role for mechanistic immune modeling, enhancing both the speed and accuracy of model calibration and validation, and, ultimately, drug development.

## FUNDING INFORMATION

No funding was received for this work.

## CONFLICT OF INTEREST

Rajat Desikan is employed by GSK and Priya Jayachandran is employed by Pfizer Inc.
